# THE DIMENSIONALITY OF FUTURE TIME PERSPECTIVE

**DOI:** 10.1093/geroni/igac059.751

**Published:** 2022-12-20

**Authors:** Daniel Grühn, Rebekah Knight

**Affiliations:** NC State University, Raleigh, North Carolina, United States; NC State University, Raleigh, North Carolina, United States

## Abstract

There have been suggestions that the measure of future time perspective shows a two-factor structure. However, the two-factor structure coincides with positively- and negatively-framed items potentially indicating a method factor rather than a content factor. By using reversed-scored items in an adult sample (N = 1421, aged 19 to 79, M = 39.1, SD = 11.1), we found evidence that the two-factor structure is mainly due to the framing of the items representing method factors rather than representing separate content factors. Item framing might be more important in aging-related research than expected.

